# Data on the administrative workload and perceived administrative burden of farmers in Switzerland

**DOI:** 10.1016/j.dib.2024.111186

**Published:** 2024-12-04

**Authors:** Gabriele Mack, Christian Ritzel, Jeanine Ammann, Katja Heitkämper, Nadja El Benni

**Affiliations:** a Agroscope, Research Group Economic Modelling and Policy Analysis, Tänikon, 8356 Ettenhausen, Switzerland; b Agroscope, Research Group Socioeconomics, Tänikon, 8356 Ettenhausen, Switzerland; c Agroscope, Research Division Sustainability Assessment and Agricultural Management, Tänikon, 8356 Ettenhausen, Switzerland

**Keywords:** Administrative burden, Agricultural policy, Policy perception, Regulatory non-compliance, Penalties, Acceptability of rules, Knowledge of rules, Administrative transaction costs

## Abstract

We present data from a paper-and-pencil survey of Swiss farmers. The survey was mailed to 2000 randomly selected Swiss farmers from the two largest Swiss language regions (German and French) in February 2019. A reminder was sent in April 2019. The response rate was around 40 % (*N* = 811). In the main part of the survey, we collected quantitative data on farmers’ workload and perceived burden due to (1) overall farming activities, (2) administrative activities related to the application of direct payments, and (3) other office work related to farm planning, bookkeeping, purchasing, and sales. We also asked farmers to rate their current workload and perceived administrative burden compared to five years earlier. We also collected data on the perceived burden of using e-government services, the administrative workload of various voluntary direct payment schemes, and the workload of inspections and sanctions. We collected personal information about the farmers. Finally, the farmers were asked to rate a series of statements regarding agricultural policy measures, the importance of inspection measures, the obligation to provide proof of eligibility for direct payments, information on current policy measures, and the justification of penalties for non-compliance with environmental or animal welfare standards. The survey results showed that, on average, Swiss farmers spent 3–5 % of their total working time on administrative tasks. The farmers rated the perceived burden of administrative activities as higher than the burden of overall farming activities or other office work. The data also showed that the farmers’ perceived administrative burden had increased compared to five years earlier. Finally, the results showed that 28 % of the Swiss farmers had received a penalty for non-compliance with direct payment regulations.

Specifications Table

**Table:** 

Subject	Social Sciences, Agricultural Economics, Public Administration
Specific subject area	Farmers’ perception of their administrative burden and non-compliance with agricultural policy regulations
Data format	Raw
Type of data	Excel file, Stata file, survey (PDF) and codebook (PFD)
Data collection	A paper-and-pencil survey of 2000 randomly selected Swiss farmers was conducted from February 2019 to April 2019. Farmers’ contact information was provided by the Swiss Federal Office for Agriculture, which maintains a database of all farm households receiving direct payments, comprising about 98 % of all Swiss farms. The farmers received a paper-and-pencil questionnaire via postal mail. The response rate was around 40 % (*N* = 808).
Data source location	Institution: Agroscope City/Town/Region: Ettenhausen, Tänikon Country: Switzerland
Data accessibility	The original dataset in Excel format (Excel and stata file, the survey in 2 languages (PDF), the English translation of the survey (PDF), and the codebook describing the variables (PDF) are freely available online on 10.5281/zenodo.12607548. Repository name: Zenodo DOI: 10.5281/zenodo.12607548
Related research article	G. Mack, C. Ritzel, J. Ammann, N. El Benni, Improving the understanding of farmers’ non-compliance with agricultural policy regulations, J. Rural Stud. 106 (2024) 103,190. 10.1016/j.jrurstud.2023.103190

## 1 Value of the Data

• In many European countries, farmers criticise the administrative burden imposed by agricultural policy regulations. These data on farmers’ assessment of their administrative workload and perceived administrative burden are important for both researchers and policymakers.
• The data on farmers’ self-reported non-compliance and the factors influencing it are important for both researchers and policymakers. Information on the reasons why farmers do not comply with direct payment regulations can help the government develop targeted measures.
• The data on farmers’ perceptions of agricultural policy help policymakers better understand farmers who support or oppose agricultural policy.
• The survey includes newly developed items to measure farmers' administrative burden, which can be replicated and modified in other settings, and further developed and validated in future studies.

## 2 Background

The shift in agricultural policy from price support to direct payments and the growing number of environmental regulations have significantly increased the administrative burden of farmers [2]. To receive payments from the state, farmers have to fill in a number of forms and compile various documents each year to prove their eligibility. However, in addition to paperwork, the direct payment system has also brought with it on-farm inspections by government authorities and fines for non-compliance [3]. In Switzerland, for example, inspections take place at least every four years, requiring farmers to provide various forms of documentation and to accompany the inspectors during the visit [1]. If non-compliance is found, the government increases the number of on-farm inspections [1]. Farmers have the option of appealing the results of the inspection, which, of course, involves additional paperwork.

Since the European Union (EU) introduced direct payments in 1992, farmers have criticised the resulting bureaucratisation of the common agricultural policy (cap) [4]. Even in 1999, an average of 61 % of eu farmers considered the administrative procedures of the cap to be ‘much too heavy’ (30 %) or ‘too heavy’ (31 %) [4]. Several studies have confirmed that farmers often perceive administrative requirements as complex, burdensome, and demanding [[5], [6], [7]]. Understanding how farmers perceive their administrative burden and what factors influence their perceptions is important for developing recommendations to further reduce administrative burden [3]. Furthermore, knowing the factors that influence non—compliance is relevant for policy and society, as non—compliance increases administrative transaction costs for authorities and farmers due to follow-up controls [1]. Based on this, we designed a study to collect a dataset that identifies and describes the factors that influence farmers’ perceived administrative burden and their non—compliance with agricultural policy rules. The related research paper [1] presents part of the data described here, focusing on non—compliance with agricultural policy regulations.

## 3 Data Description

**Fig. 1:**
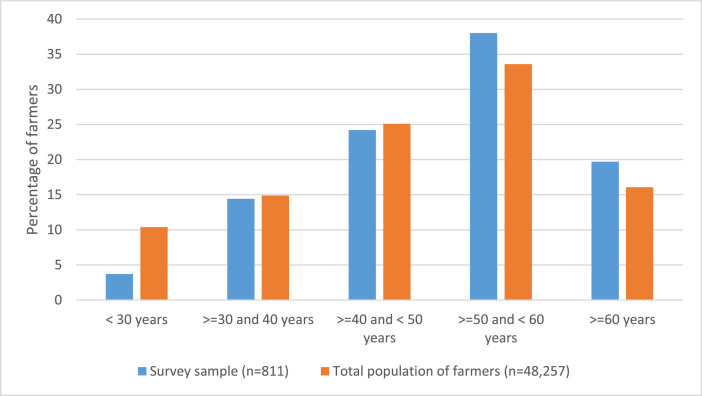
Age distributions of our survey sample compared to the total population of farmers in Switzerland. Note: The calculations for the total population are based on Swiss agricultural census (Data source: AGIS records^1^).

Our sample consisted of 811 farmers from the German and French parts of Switzerland. Table 1 shows the socio-economic characteristics of the farmers participating in the survey. In terms of age distribution, our sample is quite representative of the total population of farmers in Switzerland (Fig. 1). In the survey, we asked the farmers about their general level of education (i.e. education levels without specific agricultural training) and whether they had any agricultural training. We found that the group of farmers with a federal vocational diploma was the most represented (43 %), followed by the group with a federal professional diploma (20 %), together representing two thirds of the sample. The educational level of the survey participants and the other socio-economic variables could not be compared with the total population of farmers, as the corresponding data are not available in the Swiss Agricultural Census. Overall, 84 % of the survey participants had an agricultural education. More than 80 % of the participants had 20 years or more of farming experience. In addition, almost 90% of the farmers who took part in the survey stated that they were involved in political or agricultural organisations as board members or managers.

**Table 1:** Sample description (socio-demographic characteristics of the sample).

Socio-demographic variables	Percent	N
Age group		811
<30 years	4	
≥30 years and <40 years	14	
≥40 years and <50 years	24	
≥50 years and <60 years	38	
≥60 years	20	
General education level		789
- No vocational education	6	
- In education	0	
- Vocational education and training (VET) federal certificate	5	
- Vocational education and training (VET) federal diploma	43	
- Federal diploma of professional education and training (PET)	20	
- Advanced federal diploma of professional education and training	15	
- Higher technical college	7	
- Bachelor's, master's, or higher degree of the farm manager	4	
Agricultural training		798
- No	16	
- Yes	84	
Work experience in farming		811
<10 year	7	
≥10 years and <20 years	12	
≥20 years and <30 years	19	
≥30 years and <40 years	30	
≥40 years	32	
Off-farm work		811
No off-farm work (full-time farmers)	53.9	
<30 % off-farm work	13.2	
≥30 % and <60 % off-farm work	15.5	
≥60 % and <90 % off farm work	8.4	
≥90 % off-farm work	9.0	
Engagement as board or executive member in political or agricultural organisations		769
- Municipal council	13	
- District council	1	
- Local association	2	
- Member of executive committees of agricultural organisations	29	
- Farmers’ union board	1	
- No voluntary commitment	32	
- Other	11

The survey included questions on farmers’ workload, perceived burden, current workload compared to 5 years earlier, and perceived current burden compared to 5 years earlier. We also asked farmers to distinguish between different types of work (i.e. all farm work, administrative work, and other office work). To ensure a common understanding of the technical terms used in the questionnaire, we included a supplementary sheet with the survey describing in detail which types of work are included in all farm work, administrative work, and other office work (see Table 3 in the supplementary material). The questionnaire could be completed by farmers in 15–20 min. The questions on the farmers' workload and their administrative burden were newly developed and can be replicated and modified in other settings.

## 4 Experimental Design, Materials, and Methods

We collected survey data in Switzerland from February 2019 to April 2019. A paper-and-pencil survey was sent to 2000 randomly selected Swiss farmers. The random sample was drawn by the Swiss Federal Office for Agriculture from a database of all Swiss farm households (i.e. 98 % of the approximately 48,000 Swiss farms). No other sampling criteria or stratification methods were used to draw the random sample. The Federal Office for Agriculture also provided contact information for the farmers. About 4 weeks after the first invitation, farmers who had not responded received a reminder by post. The response rate was about 40 % (*N* = 811). A cover letter was sent as an attachment to the survey, briefly informing the farmers of the content and purpose of our study. To encourage farmers to respond to our survey, we informed them in the cover letter that all respondents would be entered into a prize draw for the chance to win one of three gift cards (worth CHF 150) from a popular Swiss retailer and that it would take about 20 min to answer the questions. We also told them that a supplementary sheet with definitions of technical terms (e.g. total farm work, administrative work, other office work) was available to help them answer the questions. We explained that their answers would be kept strictly confidential.

### 4.1 Section 1: assessment of workload and perceived burden

In this section, we asked farmers to estimate their average workload over the year in hours per week, distinguishing between any farm work, administrative work, and other office work. We then asked farmers to rate their perceived burden associated with the workload on a Likert scale from “1 = not at all onerous” to “7 = very onerous” for any farm work, administrative work, and other office work. We also asked farmers to rate the current workload for the three different types of work compared to 5 years earlier on a Likert scale from “1 = much less” to “7 = much more”. Finally, we asked farmers to rate their perceived burden of the three types of work compared to 5 years earlier on a Likert scale from “1 = much less onerous” to “7 = much more onerous.” We reminded farmers to check the definitions for total farm work, administrative work, and other office work in the supplementary sheet.

In 2019, on average over the year, the farmers spent 4.5 % of their total farm work on administrative tasks (Table 2). Assuming a 60-h working week (average over the year), this means that farmers spend on average 2.7 h per week on administrative work. For other office work, farmers spend on average 4.1 h per week. Farmers rated the perceived burden of administrative work as higher than the burden of total farm work or other office work. The descriptive results show that the time spent on administrative work and the associated perceived administrative burden increased compared to 5 years earlier. This increase was rated, on average, higher for administrative work than for farm work as a whole or for other office work.

**Table 2:** Assessment of farm work.

	Unit	M	SD	N
Average weekly workload over the year				
-Total farm work	h/week	59.6	35.1	801
-Administrative work	h/week	2.7	5.3	797
-Other office work	h/week	4.1	6.4	794
Perceived burden due to the workload				
-Total farm work	not at all onerous (1) - very onerous (7)	4.0	1.6	801
-Administrative work^a^^,^^b^	not at all onerous (1) - very onerous (7)	4.9	1.6	803
-Other office work	not at all onerous (1) - very onerous (7)	4.6	1.6	803
Current workload compared to 5 years earlier				
-Total farm work	much less (1) - much more (7)	4.5	1.2	786
-Administrative work^a^^,^^b^	much less (1) - much more (7)	5.3	1.2	786
-Other office work	much less (1) - much more (7)	5.1	1.2	786
Perceived current burden compared to 5 years earlier				
-Total farm work	much less onerous (1) - much more onerous (7)	4.4	1.2	780
-Administrative work^a^^,^^b^	much less onerous (1) - much more onerous (7)	5.1	1.3	781
-Other office work	much less onerous (1) - much more onerous (7)	4.9	1.3	779

a The mean value for administrative work is significantly higher than that for total farm work

b The mean value of administrative work is significantly higher than that of other office work.

### 4.2 Section 2: electronic data recording and internet access

**Fig. 2:**
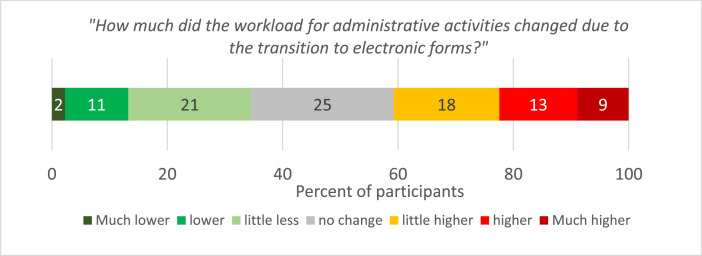
Change in workload due to the transition from written to electronic forms (*N* = 786).

**Fig. 3:**
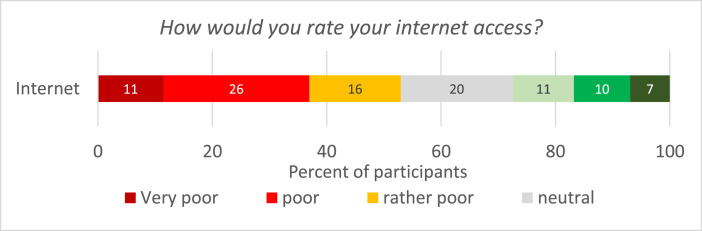
Perception of internet access (*N* = 795).

In the second part of the survey, we focused on the administrative workload resulting from the use of e-government services for applying for direct payments and internet access. For 40 % of the respondents, the switch to electronic forms increased the time spent on administrative activities. However, one-third-of respondents reported a decrease in workload (Fig. 2). On average, however, the administrative workload increased very little due to the switch to electronic forms. The majority of farmers (53 %) rated their access to the internet as poor to very poor (Fig. 3).

### 4.3 Section 3: administrative workload for the uptake of voluntary direct payment programmes

**Fig. 4:**
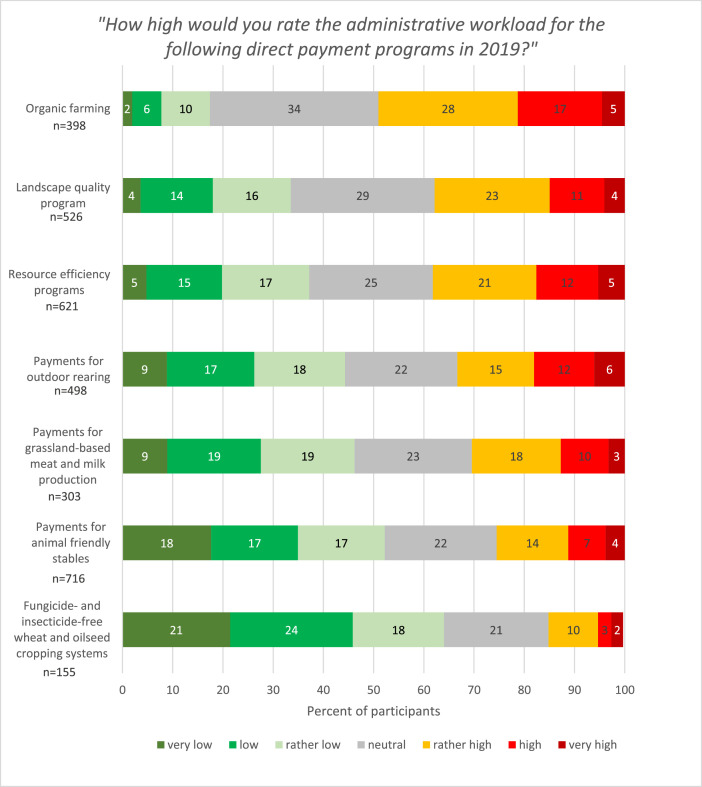
Perceived workload for administrative tasks related to voluntary direct payment programmes (subsample of participants who indicated that they had adopted the respective voluntary direct payment programme).

The administrative workload is influenced by the uptake of voluntary direct payment programmes and their associated animal welfare and environmental standards. Therefore, we included in the survey all voluntary agri-environmental and animal welfare programmes listed in the 2018 annual agricultural report of the Swiss Federal Office of Agriculture and asked farmers which of them they had adopted [8]. We also used the definitions of voluntary direct payment programmes in the annual agricultural report to describe the programmes in the survey [8]. We asked farmers to rate their administrative workload on a seven-point Likert scale from “1 = very low” to “7 = very high” for their adopted voluntary direct payment programmes (Fig. 4). The results of the survey showed that the agro-environmental programme for fungicide- and insecticide-free wheat and oilseed production systems (the so-called Extenso programme) is associated with the lowest administrative workload. Overall, 64 % of the respondents indicated that the workload required was low to very low. Participation in the animal welfare programme for animal-friendly housing systems was also associated with a low administrative workload, whereas adoption of organic farming and landscape quality programmes was associated with the highest administrative workload.

### 4.4 Section 4: administrative workload due to on-farm inspections

Specific administrative activities, such as the calculation of a nutrient balance at the farm level and direct payment inspections, are often criticised by farmers for their perceived high workload. We therefore asked farmers to estimate the time spent on the following four administrative activities (Table 3):

(1) Calculating the nutrient balance (necessary to receive direct payments).
(2) Making all documents available for direct payment inspections.
(3) Attending on-farm inspections.
(4) Filing a complaint following on-farm inspections.

**Table 3:** Administrative workload for inspections.

	Percent	N
Workload for provision of necessary documents for inspections		797
- Less than 2 h per inspection	36	
- 2 – less than 4 h per inspection	40	
- 4 – less than 6 h per inspection	15	
- 6 h or more per inspection	9	
Required time for accompanying on-farm inspections		799
- Less than 0.5 h	1	
- 0.5 – under 1 h	9	
- 1 – under 1.5 h	24	
- 1.5 – under 2 h	30	
- 2 – under 2.5 h	20	
- Over 2.5 h	17	
Complaints as a result of on-farm inspections		802
- Yes	13	
- No	87	
Have you ever received a penalty because of non-compliance with direct payment rules?		801
- Yes	28	
- No	72	
Time spent on complaints due to direct payment inspections		553
- Less than 30 min	67	
- 30 – under 60 min	17	
- 60 – under 90 min	5	
- 90 – under 120 min	4	
- 120 – under 150 min	2	
- 150 min or more	5	

**Fig. 5:**
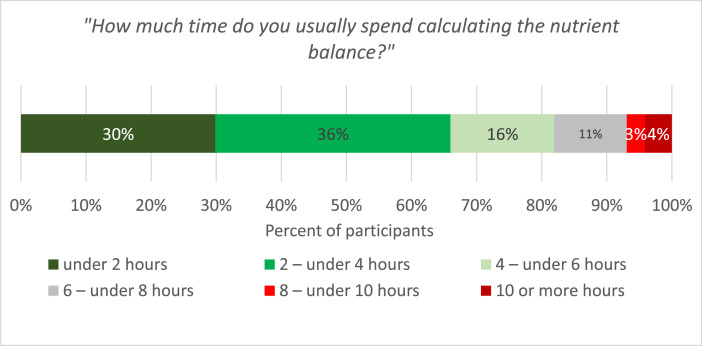
Time required for the calculation of the nutrient balance (subsample of farmers who indicated that they calculated the nutrient balance by themselves *N* = 144).

We found that 82 % of the farmers had the SUISSE balance sheet filled in by an agricultural adviser, a farmer they knew, a feed supplier, or another person. Among the participants, 18 % calculated the nutrient balance by themselves. About two-thirds of this subsample need less than 6 h to calculate the nutrient balance (Fig. 5). The majority of the respondents (76 %) needed less than 4 h to prepare all the necessary documents for inspections, and less than 64 % needed less than 2 h to accompany inspectors during the on-farm visit.

Furthermore, we asked farmers whether they had ever received a penalty for non-compliance with direct payment regulations and whether they had ever submitted a complaint (Table 3).

A minority of 13 % had lodged a complaint as a result of an inspection, while 28 % of the participants had received a penalty for non-compliance with direct payment regulations (Table 3).

### 4.5 Section 5: personal information on participants

See Table 1.

### 4.6 Section 6: statements on agricultural policy

**Fig. 6:**
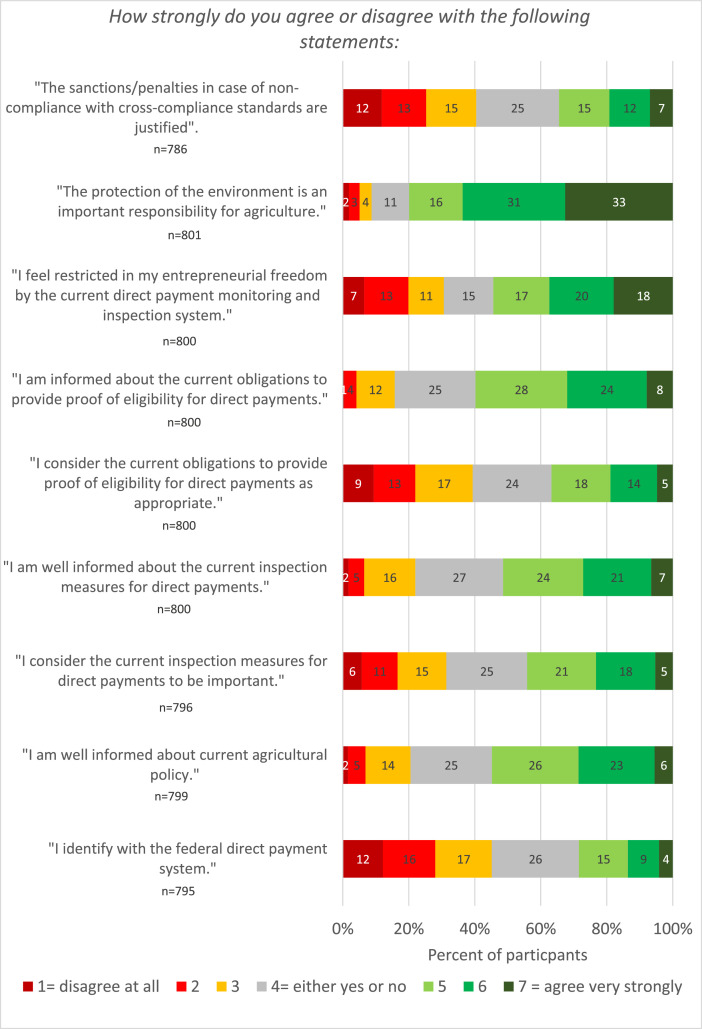
Percentage of participants who agree/disagree with the statements on agricultural policy.

Only a minority of 28 % of the participants agreed with the statement “*I identify with the federal direct payment system*”, while 45 % of the participants disagreed with this statement (see Fig. 6). A majority of the participants stated that they were well informed about current agricultural policy. Furthermore, about half of the participants (52 %) agreed with the statement “*I am well informed about the current inspection measures*”, and 60 % of the participants agreed with the statement “*I am informed about the current obligations to provide proof of eligibility for direct payments*”.

However, acceptance of the policy measures was low. A minority of 44 % agreed with the statement “*I consider the current inspection measures for direct payments to be important*” (Fig. 6), and a minority of 37 % agreed with the statement “*I consider the current obligations to provide proof of eligibility for direct payments to be appropriate”.* A majority of 55 % agreed with the statement *“I feel restricted in my entrepreneurial freedom by the current direct payment monitoring and inspection system”*. Acceptance of penalties for non-compliance with direct payment regulations was also low, with a minority of 34 % agreeing that penalties for non-compliance with regulations were justified.

### 4.7 Section 7: open text box and end of the survey

Farmers were given the opportunity to write their opinions regarding the agricultural policy. The following statements show that farmers found the daily record-keeping obligations particularly burdensome, as they were easily forgotten and difficult to integrate into their daily work routine. In addition, the constant innovations and the many complex regulations were perceived as burdensome:

- “The problem is not the time for administrative work, but the fact that almost every day there is something to remember, crosses in journals, TVD reports, etc.”
- “In summer, after the harvest and stable work (up to 16 h), I find it stressful to enter everything so that everything is always up to date. But I really enjoy working with animals, plants, and machines.
- “The worst thing is the constant pressure to keep records. The problem is that it is often not possible to keep records while working and then they have to be updated”.
- “The administrative work on my farm wouldn't be too burdensome in itself. The only burden is that I often have too little time for office work and other work (development and maintenance tasks) in addition to the work in the barn and outside”.
- “In addition to the administrative work, there is also the mental workload or stress. This is always forgotten. It's very high for me. When my first thoughts every morning are: Have I updated all the records, can an inspector come to the farm today, is everything in order because yesterday was a busy day? In the case of a registered inspection, do I have all the documents, is everything in order, what happens if something is not quite right?
- “Unfortunately, the constant changes and innovations in the Suisse balance, for example, create a lot of additional administrative work. It also means that the control and enforcement bodies are completely overwhelmed, not to mention us farmers who have other worries every day with our daily work with animals, crops, etc.”
- “The administration always worries me before and during inspections. It's the uncertainty, because everything changes very quickly and is different every year”.
- “Get rid of the Suisse balance sheet—useless paper tiger. It's difficult to keep track of all the regulations (LQB, networking, BFF2, BTS, Raus, Bio, Extenso, etc.)”.
- “Please don't make more regulations. The farmer can no longer keep up. It's too much; it's unmanageable. I have a lot of conversations about overload. Young people say there are too many regulations.
- “All the documents that need to be recorded should be sent out every year. So you don't have to chase after documents and be afraid of forgetting something!”
- “In principle, it could be simpler. Unfortunately, I often don't understand what is meant, or I feel insecure and have to ask specialists, which is sometimes time-consuming. The person in charge is not available. I have to make several phone calls”.
- “IP Swiss points, Q2 contributions, TVD, animal health journal, medication sheet, alcohol administration, etc. Endless documentation of regulations. The chance of missing something, missing a deadline, or not being up to date is enormous. You can spend days just reading the newsletter. The likelihood of not being able to use the DZ to the full is increasing; the changes are happening far too fast. Is this intentional?”
- “The administrative effort is high, because no mistakes are allowed and you have to send it or confirm it. Maintaining good software instead of constantly changing the interface would save a lot of effort!”

## Limitations

A limitation of our research is that we did not consider the different subject matters of non-compliance (i.e. incorrect carry-over of a value and use of a chemical in excess of the permitted limit). Consequently, we could not link these topics to the factors included in our research. However, this might be a starting point for further research.

## Ethics Statement

The researchers adhered to all ethical considerations during the data collection process and followed institutional guidelines [9]. Participation was voluntary. The participants were assured full anonymity and data privacy in the cover letter of the survey.

## Credit Author Statement

**Gabriele Mack:** Conceptualisation, Investigation, Data curation, Writing-Original draft – Reviewing and Editing, Project administration. **Christian Ritzel:** Methodology, Investigation, Writing – Reviewing and editing. **Jeanine Ammann:** Conceptualisation, Methodology, Writing – Reviewing and editing. **Katja Heitkämper:** Conceptualisation, Data curation, Writing – Reviewing and editing. **Nadja ElBenni:** Conceptualisation, Methodology, Writing – Reviewing and Editing, Resources.

## Data Availability

ZenodoData on the administrative workload and perceived administrative burden of farmers in Switzerland (Original data).

## Appendix Supplementary materials

Image, application 1

## Footnotes

1 See https://www.blw.admin.ch/blw/de/home/politik/datenmanagement/agate/agis.html
