# Supplementary material for: Data on the administrative workload and perceived administrative burden of farmers in Switzerland
Source: Data Brief. 2024 Dec 4;58:111186. doi: 10.1016/j.dib.2024.111186 (PMC11867125; doi:10.1016/j.dib.2024.111186)
Supplement: Supplementary file 1 [file mmc1.docx]

**Supplementary material**

**Table 1**

Survey in German (original) and its English translation

| No | **German (original)** | English translation |
| --- | --- | --- |
| **1. Einschätzung ihres Arbeitsaufwandes** | | **1. Estimation of your workload** |
| 1.1 | Im Durchschnitt über das ganze Jahr verteilt: Wie hoch schätzen Sie Ihren gesamten Arbeitsaufwand auf Ihrem Betrieb pro Woche? (siehe Beiblatt Begriffserläuterung)  Durchschnittlicher wöchentlicher  Arbeitsaufwand in Stunden pro Woche | Average over the year: What is your estimate of the total weekly workload on your farm? (See supplementary sheet for an explanation of terms.)  Average weekly workload in hours per week? |
| 1.2 | Im Durchschnitt über das ganze Jahr verteilt: Wie hoch schätzen Sie Ihren Aufwand für administrative Tätigkeiten im Zusammenhang mit Direktzahlungen pro Woche? (siehe Beiblatt Begriffserläuterung)  Durchschnittlicher wöchentlicher  Arbeitsaufwand für administrative Tätigkeiten in Stunden pro Woche | Average over the year: What is your estimate of your workload per week for administrative work related to direct payments? (See supplementary sheet for an explanation of terms.)  Average weekly administrative workload in hours per week? |
| 1.3 | Im Durchschnitt über das ganze Jahr verteilt: Wie hoch schätzen Sie Ihren Aufwand für Büroarbeiten pro Woche? (siehe Beiblatt Begriffserläuterung)  Durchschnittlicher wöchentlicher  Arbeitsaufwand für Büroarbeiten in Stunden pro Woche | On average over the whole year: How much do you estimate your workload for other office work per week? (See supplementary sheet for explanation of terms.)  Average weekly workload for office work in hours per week? |
| 1.4 | Wie belastend stufen Sie den jeweiligen Arbeitsaufwand ein? (siehe Beiblatt Begriffserläuterung)  Überhaupt nicht belastend (1) – sehr belastend (7)   - Gesamter Arbeitsaufwand - Arbeitsaufwand administrative Tätigkeiten - Arbeitsaufwand Büroarbeiten | How onerous would you rate your current workload? (See supplementary sheet for explanation of terms.)  Not at all onerous (1) – Very onerous (7)   - Total farm work - Administrative work - Other office work |
| 1.5 | Wie ist der jeweilige Arbeitsaufwand heute im Vergleich zu der Zeit vor 5 Jahren? (siehe Beiblatt Begriffserläuterung)  Sehr viel geringer (1) – sehr viel höher (7)   - Gesamter Arbeitsaufwand - Arbeitsaufwand administrative Tätigkeiten - Arbeitsaufwand Büroarbeiten | How is your workload now compared to 5 years ago? (See supplementary sheet for definitions.)  Much less (1) – Much more (7)   - Total farm work - Administrative work - Other office work |
| 1.6 | Wie belastend ist der jeweilige Arbeitsaufwand heute im Vergleich zu der Zeit vor 5 Jahren? (siehe Beiblatt Begriffserläuterung)  Sehr viel weniger belastend (1)- sehr viel mehr belastend (7)   - Gesamter Arbeitsaufwand - Arbeitsaufwand administrative Tätigkeiten - Arbeitsaufwand Büroarbeiten | How onerous is the workload now compared to 5 years ago? (See supplementary sheet for definition of terms.)  Much less onerous (1) – much more onerous (7)   - Total farm work - Administrative work - Other office work |
| 1. **Elektronische Datenerfassung** | | 1. Electronic data recording |
| 2.1 | Who creates the GIS parcel plans?  -Eigener Betrieb  -Landwirtschaftsamt | Who creates the GIS parcel plans?  - Own farm  - Cantonal agricultural office |
| 2.2 | Benötigen Sie Unterstützung durch Dritte, um GIS Parzellenpläne zu erstellen?  -Ja  -Nein | Do you need third-party support to create GIS plot plans?  - Yes  - No |
| 2.3 | Beantragung von Direktzahlungen: Wie stark hat sich der administrative Arbeitsaufwand durch den Wechsel auf elektronische Formulare verändert?  Sehr viel geringer geworden (1)- sehr viel höher geworden (7)  -Arbeitsaufwand administrative Tätigkeiten | Applying for direct payments: How much did the workload for administrative tasks change due to the transition to electronic forms?  Much lower (1) – much higher (7)   - Workload for administrative activities |
| 2.4 | Wie beurteilen Sie Ihren Internet-Zugang?  Sehr schlecht (1) – sehr gut (7) | How would you rate you internet access?  Very bad (1) – very good (7) |
| 1. **Freiwillige Direktzahlungsprogramme** | | 1. **Voluntary agri-environmental programmes** |
| 3.1 | **Beiträge für den biologischen Landbau** | **Payments for organic farming** |
|  | Bewirtschaften Sie Ihren Betrieb nach den Richtlinien des biologischen Landbaus?  -ja  -nein | Do you manage your farm in accordance with organic farming guidelines?  - Yes  - No |
|  | Wenn ja, wie hoch stufen Sie den Arbeitsaufwand für administrative Tätigkeiten in 2019 dafür ein?  Sehr gering (1) – sehr hoch (7) | If yes, what is your estimate of the administrative workload for organic farming in 2019?  Very low (1) – very high (7) |
| 3.2 | **Landschaftsqualitätsbeiträge** | **Landscape quality payments** |
|  | Nehmen Sie an diesem Direktzahlungsprogramm teil?  -ja  -nein | Do you participate in this direct payment program?  - Yes  - No |
|  | Wenn ja, wie hoch stufen Sie den Arbeitsaufwand für administrative Tätigkeiten in 2019 dafür ein?  Sehr gering (1) – sehr hoch (7) | If yes, how high would you rate the workload for administrative tasks in 2019 for the adoption of the landscape quality program?  Very low (1) – very high (7) |
| 3.3 | **Beitrag für extensives Getreide, Raps etc. (Extenso)** | **Payments for fungicide and insecticide-free wheat and rapeseed (Extenso)** |
|  | Nehmen Sie an diesem Direktzahlungsprogramm teil?  -ja  -nein | Do you participate in this direct payment program?  - Yes  - No |
|  | Wenn ja, wie hoch stufen Sie den Arbeitsaufwand für administrative Tätigkeiten in 2019 dafür ein?  Sehr gering (1) – sehr hoch (7) | If yes, how high would you rate the workload for administrative tasks in 2019 for the adoption of fungicide- and insecticide-free wheat and rapeseed?  Very low (1) – very high (7) |
| 3.4 | **Tierwohlbeiträge (BTS)** | **Payments for animal friendly stables** |
|  | Nehmen Sie an diesem Direktzahlungsprogramm teil?  -ja  -nein | Do you participate in this direct payment program?  - Yes  - No |
|  | Wenn ja, wie hoch stufen Sie den Arbeitsaufwand für administrative Tätigkeiten in 2019 dafür ein?  Sehr gering (1) – sehr hoch (7) | If so, how high would you rate the workload for administrative tasks in 2019 for the adoption of the programme for animal friendly stables?  Very low (1) – very high (7) |
| 3.5 | **Tierwohlbeiträge (RAUS)** | **Payments for outdoor rearing** |
|  | Nehmen Sie an diesem Direktzahlungsprogramm teil?  -ja  -nein | Do you participate in this direct payment program?  - Yes  - No |
|  | Wenn ja, wie hoch stufen Sie den Arbeitsaufwand für administrative Tätigkeiten in 2019 dafür ein?  Sehr gering (1) – sehr hoch (7) | If yes, how high would you rate the workload for administrative tasks in 2019 for the adoption of the programme for outdoor rearing?  Very low (1) – very high (7) |
| 3.6 | **Beitrag für graslandbasierte Milch- und Fleischproduktion (GMF)** | **Payments for grassland-based milk and meat program** |
|  | Nehmen Sie an diesem Direktzahlungsprogramm teil?  -ja  -nein | Do you participate in this direct payment program?  - Yes  - No |
|  | Wenn ja, wie hoch stufen Sie den Arbeitsaufwand für administrative Tätigkeiten in 2019 dafür ein?  Sehr gering (1) – sehr hoch (7) | If so, how high would you rate the workload for administrative tasks in 2019 for the adoption of the grassland-based milk and meat program?  Very low (1) – very high (7) |
| 3.7 | **Ressourceneffizienzprogramme** | **Resource efficiency programmes** |
|  | Nehmen Sie an diesem Direktzahlungsprogramm teil?  -ja  -nein | Do you participate in this direct payment program?  - Yes  - No |
|  | Wenn ja, wie hoch stufen Sie den Arbeitsaufwand für administrative Tätigkeiten in 2019 dafür ein?  Sehr gering (1) – sehr hoch (7) | If so, how high would you rate the workload for administrative tasks in 2019 for the adoption of the resource efficiency program?  Very low (1) – very high (7) |
| 1. **Administrativer Aufwand für Direktzahlungskontrollen** | | 1. **Administrative workload for inspections** |
| 4.1 | Wie lange brauchen Sie normalerweise im Jahr, um alle erforderlichen Dokumente für die Direktzahlungskontrollen bereitzustellen (Nährstoffbilanz, Parzellenjournals, Fruchtfolgepläne, Auslaufjournals, Wiesenkalender/Wiesenjournal, Feldkalender/ Kulturblätter, Inventar Zukauf von Pflanzenschutzmittel/Dünger, etc.)?  - unter 20 Minuten  - 20 – unter 40 Minuten  - 40 – unter 60 Minuten  - 60 – unter 80 Minuten  - 80 – unter 100 Minuten  - 100 – unter 120 Minuten  - 120 – unter 140 Minuten  - 140 – unter 160 Minuten  - 160 – unter 180 Minuten  - 180 Minuten oder mehr | How long do you normally need each year to provide all the documents required for direct payment inspections (nutrient balance, plot journals, crop rotation plans, field journals, meadow calendars/meadow journals, field calendars/crop sheets, inventory of purchased pesticides/fertilisers, etc.)?  - Less than 20 minutes  - 20 – less than 40 minutes  - 40 – less than 60 minutes  - 60 – less than 80 minutes  - 80 – less than 100 minutes  - 100 – less than 120 minutes  - 120 – less than 140 minutes  - 140 – less than 160 minutes  - 160 – less than 180 minutes  - 180 minutes or more |
| 4.2 | Berechnen Sie die SUISSE-Bilanz selbst?   - Ja - Nein | Do you calculate the SUISSE balance sheet by yourself?   - Yes - No |
| 4.3 | Wenn ja, welche Aufzeichnungsform verwenden Sie für die SUISSE-Bilanz?   - Formular auf Papier - Online-Formular - Software | If so, which recording type do you use for the SUISSE balance sheet?  - Paper form  - Online form  - Software |
| 4.4 | Wenn ja, wie gross ist normalerweise Ihr Zeitaufwand für die Berechnung der SUISSE-Bilanz?   - unter 2 Stunden - 2 – unter 4 Stunden - 4 – unter 6 Stunden - 6 – unter 8 Stunden - 8 – unter 10 Stunden - 10 oder mehr Stunden | If so, how much time do you normally spend calculating the SUISSE balance sheet?  - under 2 hours  - 2 – under 4 hours  - 4 – under 6 hours  - 6 – under 8 hours  - 8 – under 10 hours  - 10 or more hours |
| 4.5 | Wie hoch ist normalerweise Ihr Zeitaufwand, um alle Unterlagen für die Direktzahlungskontrolle bereitzustellen?  - unter 2 Stunden je Kontrolle  - 2 – unter 4 Stunden je Kontrolle  - 4 – unter 6 Stunden je Kontrolle  - 6 Stunden oder mehr je Kontrolle | How much time do you spend to provide all the necessary documents for the inspections?  - Less than 2 hours per inspection  - 2 – less than 4 hours per inspection  - 4 – less than 6 hours per inspection  - 6 hours or more per inspection |
| 4.6 | Wie lange sind Sie normalerweise bei einer Direktzahlungskontrolle in Ihrem Betrieb anwesend?  -unter 0.5 Stunden  -0.5 – unter 1 Stunden  - 1 – unter 1.5 Stunden  - 1.5 – unter 2 Stunden  - 2 – unter 2.5 Stunden  - über 2.5 Stunden | How much time does it require to accompany on-farm inspections?  - Less than 0.5 hours  - 0.5 – under 1 hour  - 1 – under 1.5 hours  - 1.5 – under 2 hours  - 2 – under 2.5 hours  - Over 2.5 hours |
| 4.7 | Waren Sie in den letzten drei Jahren mit den Ergebnissen einer Direktzahlungskontrolle unzufrieden und haben reklamiert?   - Ja - Nein | Have you been dissatisfied with the results of a direct payment inspection in the last three years and made a complaint?  - Yes  - No |
| 4.8 | Wurden Sie im Zusammenhang mit Direktzahlungskontrollen jemals gebüsst?   - Ja - Nein | Have you ever received a penalty in the context of direct payment inspections?  - Yes  - No |
| 4.9 | Wie hoch war Ihr Zeitaufwand für Reklamationen im Zusammenhang mit einer Direktzahlungskontrolle (Telefonate, Beratungsgespräche, etc.)?   - unter 30 Minuten - 30 – unter 60 Minuten - 60 – unter 90 Minuten - 90 – unter 120 Minuten - 120 – unter 150 Minuten - 150 Minuten oder mehr | How much time did you spend on complaints in connection with a direct payment inspections (telephone calls, consultations, etc.)?  - Less than 30 minutes  - 30 – under 60 minutes  - 60 – under 90 minutes  - 90 – under 120 minutes  - 120 – under 150 minutes  - 150 minutes or more |
| 1. **Persönliche Angaben** | | 1. **Personal details** |
| 5.1 | Welches ist Ihre höchste abgeschlossene berufliche Ausbildung?  1)keine Berufsausbildung  2)in Ausbildung/Lehre begonnen  3)Berufliche Grundbildung EBA  4) Berufliche Grundbildung EFZ  5) Berufsprüfung BP  6) Höhere Fachprüfung HFP  7) Höhere Fachschule  8) Bachelor/Master oder höher | What is the highest level of vocational training you have completed?  1) No vocational education  2) In education  3) Vocational education and training (VET) federal certificate  4) Vocational education and training (VET) federal diploma  5) Federal diploma of professional education  and training (PET)  6) Advanced federal diploma of professional education and training  7) Higher technical college  8) Bachelor’s, master’s, or higher degree |
| 5.2 | Besitzen Sie eine landwirtschaftliche Ausbildung?  -Ja  -Nein | Do you have agricultural training?  - Yes  - No |
| 5.3 | Seit wie vielen Jahren arbeiten Sie in der Landwirtschaft?  Years | How many years have you been working in agriculture?  Years |
| 5.4 | Führen Sie als Betriebsleiter/Betriebsleiterin Ihren Betrieb im Nebenerwerb?   - Ja, im Nebenerwerb zu …. Prozent - Nein | As a farm manager, do you manage your farm as a part-time farm?  - Yes, as a part-time farm to xy percent  - No |
| 5.5  5.6 | Gehen Sie regelmässig beruflich einer ausserlandwirtschaftlichen Tätigkeit(en) nach?   - Ja - Nein   Wenn ja, mit welchem Pensum betreiben Sie die ausserlandwirtschaftlich(en) Tätigkeit(en)?   - Prozent Pensum | Do you regularly engage in off-farm work?  - Yes  - No  If yes, what is your percentage of off-farm work?   - Percentage |
| 5.7 | Waren Sie jemals ehrenamtlich in der Politik oder im Bereich Landwirtschaft engagiert? (Mehrere Antworten möglich)   - Bei der Gemeinde - Beim Kanton - In einem Verein - In einem Fachgremium - Beim Bauernverband - Ich hatte noch nie ein Ehrenamt - Sonstige: | Have you ever been active as board or executive member in political or agricultural organisations? (Multiple answers possible):   - Municipal council - District council - Local association - Member of executive committees of agricultural organisations - Farmers’ union board - No voluntary commitment - Other: |
| 5.8 | Welches Alter haben Sie? | What is your age? |
| 5.9 | Wie stark treffen die folgenden Aussagen für Sie zu?  Überhaupt nicht (1) – sehr stark (7)   - Ich identifiziere mich mit dem Direktzahlungssystem des Bundes. - Ich bin über die gegenwärtige Agrarpolitik gut informiert. - Ich halte die gegenwärtigen Kontrollmassnahmen für die Direktzahlungen für wichtig. - Ich bin über die gegenwärtigen Kontrollmassnahmen für die Direktzahlungen gut informiert. - Ich halte die gegenwärtigen Pflichten zur Aufzeichnung von Betriebsdaten für richtig. - Ich bin über gegenwärtige Pflichten zur Aufzeichnung von Betriebsdaten informiert. - Ich fühle mich durch die Direktzahlungskontrollen in meinem unternehmerischen Freiraum eingeschränkt. - Der Schutz der Umwelt ist eine wichtige Aufgabe der Landwirtschaft. - Die Sanktionen/Bussen bei einer Verletzung der ÖLN-Auflagen sind gerechtfertigt. | How strongly do you agree or disagree with the following statements?  Disagree at all (1) – agree very strongly (7)   - I identify with the federal direct payment system. - I am well informed about current agricultural policy. - I consider the current inspection measures for direct payments to be important. - I am well informed about the current inspection measures for direct payments. - I consider the current obligations to provide proof of eligibility for direct payments appropriate. - I am well informed about the current obligations to provide proof of eligibility for direct payments. - I feel restricted in my entrepreneurial freedom by the current direct payment monitoring and inspection system. - The protection of the environment is an important responsibility for agriculture. - The sanctions/penalties in the case of non-compliance with cross-compliance standards are justified. |

**Table 2**

Coding of the survey and English translation of the survey

| No. | English translation of the survey | **Coding** |
| --- | --- | --- |
| 1.1 | Average over the year: What is your estimate of the total weekly workload on your farm? (See supplementary sheet for explanation of terms.)  Average weekly workload in hours per week? | GesArbeitsaufwand_11 |
| 1.2 | Average over the year: What is your estimate of your workload per week for administrative tasks related to direct payments? (See supplementary sheet for explanation of terms.)  Average weekly administrative workload in hours per week? | Admin_Arbeit_12 |
| 1.3 | On average over the whole year: How much do you estimate your workload for other office activities per week? (See supplementary sheet for explanation of terms.)  Average weekly workload for office activities in hours per week? | Bueroarbeit_13 |
| 1.4 | How onerous would you rate your current workload? (See supplementary sheet for explanation of terms.)  Not at all onerous (1) – Very onerous (7) |  |
|  | - Total farm work | GesArbeitsaufwand_14 |
|  | - Administrative work | Admin_Arbeit_14 |
|  | - Other office work | Bueroarbeit_14 |
| 1.5 | How is your workload now compared to 5 years ago? (See supplementary sheet for definitions.)  Much less (1) – Much more (7) |  |
|  | - Total farm work | GesArbeitsaufwand_15 |
|  | - Administrative work | Admin_Arbeit_15 |
|  | - Other office work | Bueroarbeit_15 |
| 1.6 | How onerous is the workload now compared to 5 years ago? (See supplementary sheet for definition of terms.)  Much less onerous (1) – much more onerous (7) |  |
|  | - Total farm work | GesArbeitsaufwand_16 |
|  | - Administrative work | Admin_Arbeit_16 |
|  | - Other office work | Bueroarbeit_16 |
| 2.1 | Who creates the GIS parcel plans?  - Own farm [1]  - Cantonal agricultural office [0] | GIS_Plan_21 |
| 2.2 | Do you need third-party support to create GIS plot plans?  - Yes [1]  - no [0] | GIS_Unterstuetzung_22 |
| 2.3 | Applying for direct payments: How much did the workload for administrative tasks change due to the transition to electronic forms?  Much lower (1) – much higher (7)  Workload for administrative activities | Admin_Aufwand_23 |
| 2.4 | How would you rate your internet access?  Very bad (1) – very good (7) | Internet_24 |
| 3.1 | What is your estimate of the administrative workload for the adoption of organic farming in 2019?  Very low workload (1)  Low workload (2)  Slightly low workload (3)  Neither low nor high workload (4)  Slightly high workload (5)  High workload (6)  Very high workload (7)  No adoption of organic farming (0) | Bio_31 |
| 3.2 | What is your estimate of the administrative workload for the adoption of the landscape quality programme in 2019?  Very low workload (1)  Low workload (2)  Slightly low workload (3)  Neither low nor high workload (4)  Slightly high workload (5)  High workload (6)  Very high workload (7) | Landschaftsqualitaets_32 |
|  | No landscape quality programme (0) |  |
| 3.3 | What is your estimate of the administrative workload for the adoption of fungicide- and insecticide-free wheat and rapeseed in 2019?  Very low workload (1)  Low workload (2)  Slightly low workload (3)  Neither low nor high workload (4)  Slightly high workload (5)  High workload (6)  Very high workload (7)  No fungicide- and insecticide-free wheat and rapeseed (0) | Extenso_33 |
| 3.4 | What is your estimate of the administrative workload for the adoption of the animal friendly stable programme in 2019?  Very low workload (1)  Low workload (2)  Slightly low workload (3)  Neither low nor high workload (4)  Slightly high workload (5)  High workload (6)  Very high workload (7)  No adoption of the animal friendly stable programme (0) | BTS_34 |
| 3.5 | What is your estimate of the administrative workload for the adoption of the outdoor rearing programme in 2019?  Very low workload (1)  Low workload (2)  Slightly low workload (3)  Neither low nor high workload (4)  Slightly high workload (5)  High workload (6)  Very high workload (7)  No adoption of the outdoor rearing programme (0) | RAUS_35 |
| 3.6 | What is your estimate of the administrative workload for the adoption of the grassland-based milk and meat programme in 2019?  Very low workload (1)  Low workload (2)  Slightly low workload (3)  Neither low nor high workload (4)  Slightly high workload (5)  High workload (6)  Very high workload (7)  No adoption of the grassland-based milk and meat programme (0) | GMF_36 |
| 3.7 | What is your estimate of the administrative workload for the adoption of the resource efficiency programme in 2019?  Very low workload (1)  Low workload (2)  Slightly low workload (3)  Neither low nor high workload (4)  Slightly high workload (5)  High workload (6)  Very high workload (7)  No adoption of the resource efficiency programme (0) | Ressourcen_37 |
| 4.1 | How long do you normally need each year to provide all the documents required for direct payment inspections (nutrient balance, plot journals, crop rotation plans, field journals, meadow calendars/meadow journals, field calendars/crop sheets, inventory of purchased pesticides/fertilisers, etc.)?  - Less than 20 minutes (10)  - 20 – less than 40 minutes (30)  - 40 – less than 60 minutes (50)  - 60 – less than 80 minutes (70)  - 80 – less than 100 minutes (90)  - 100 – less than 120 minutes (110)  - 120 – less than 140 minutes (130)  - 140 – less than 160 minutes (150)  - 160 – less than 180 minutes (170)  - 180 minutes or more (190) | Unterlagen_Kontrollen_41 |
| 4.2 4.3 | Do you calculate the SUISSE balance sheet by yourself? If so, which recording type do you use for the SUISSE balance sheet?   \|  \| No calculation of the SUISSE balance (0) \| \| \| --- \| --- \| --- \| \|  \| Yes, using paper forms (1) \|  \| \|  \| Yes, using online forms (2) \|  \| \|  \| Yes, using software (3) \|  \| | SWISSBILANZ_423 |
| 4.2 4.4 | Do you calculate the SUISSE balance sheet by yourself? If so, how much time do you normally spend calculating the SUISSE balance sheet?  - No calculation of the SUISSE Balance (0)  - Under 2 hours (1)  - 2 – under 4 hours (3)  - 4 – under 6 hours (5)  - 6 – under 8 hours (7)  - 8 – under 10 hours (9)  - 10 or more hours (11) | SWISSBILANZ_44 |
| 4.5 | How much time do you spend to provide all the necessary documents for the inspections?  - Less than 2 hours per inspection (1)  - 2 – less than 4 hours per inspection (3)  - 4 – less than 6 hours per inspection (5)  - 6 hours or more per inspection (7) | Unterlagen_DK_45 |
| 4.6 | How much time does it require to accompany on-farm inspections?  - Less than 0.5 hours (15)  - 0.5 – under 1 hour (45)  - 1 – under 1.5 hours (75)  - 1.5 – under 2 hours (105)  - 2 – under 2.5 hours (135)  - Over 2.5 hours (165) | Anwesen_DK_46 |
| 4.7 | Have you been dissatisfied with the results of a direct payment inspection in the last three years and made a complaint?  - Yes (1)  - No (0) | Reklamationen_47 |
| 4.8 | Have you ever received a penalty in the context of direct payment inspections?  - Yes (1)  - No (0) | Busse_48 |
| 4.9 | How much time did you spend on complaints in connection with a direct payment inspections (telephone calls, consultations, etc.)?  - Less than 30 minutes (15)  - 30 – under 60 minutes (45)  - 60 – under 90 minutes (75)  - 90 – under 120 minutes (105)  - 120 – under 150 minutes (135)  - 150 minutes or more (165) | Zeit_Reklamationen_49 |
| 5.1 | What is the highest level of vocational training you have completed?  - No vocational education (0)  - In education (1)  - Vocational education and training (VET) federal certificate (2)  - Vocational education and training (VET) federal diploma (3)  - Federal diploma of professional education  and training (PET) (4)  - Advanced federal diploma of professional education and training (5)  - Higher technical college (6)  - Bachelor’s, master’s, or higher degree (7) | Ausbildung_51 |
| 5.2 | Do you have agricultural training?  - Yes (1)  - No (0) | LandwAusbildung_52 |
| 5.3 | How many years have you been working in agriculture?  Years | JahreAusbildung_53 |
| 5.5  5.6 | Do you regularly engage in off-farm work?  - Yes (1)  - No (0)  If yes, what is your percentage of off-farm work?   - Percentage | Nebenerwerb_55  Ausserland_556 |
| 5.7 | Have you ever been active as board or executive member in political or agricultural organisations?   - Municipal council (1) - District council (2) - Local association (3) - Member of executive committees of agricultural organisations (4) - Farmers’ union board (5) - No voluntary commitment (0) - Other (6) | Ehrenamt_57 |
| 5.8 | What is your age? | Alter_58 |
| 5.9 | How strongly do you agree or disagree with the following statements?  Disagree at all (1) – agree very strongly (7) |  |
|  | - I identify with the federal direct payment system. | Direktzahlungssystem_591 |
|  | - I am well informed about current agricultural policy. | Agrarpolitik_592 |
|  | - I consider the current inspection measures for direct payments to be important. | Kontrollen_593 |
|  | - I am well informed about the current inspection measures for direct payments. | Kontrollen_Info_594 |
|  | - I consider the current obligations to provide proof of eligibility for direct payments to be appropriate. | Aufzeichungspflicht_Richtig_595 |
|  | - I am informed about the current obligations to provide proof of eligibility for direct payments. | Aufzeichungspflicht_Info_596 |
|  | - I feel restricted in my entrepreneurial freedom by the current direct payment monitoring and inspection system. | Unternehm_Freiraum_597 |
|  | - The protection of the environment is an important responsibility for agriculture. | Umwelt_598 |
|  | - The sanctions/penalties in case of non-compliance with cross-compliance standards are justified. | Bussen_599 |

**Table 3**

Definitions of the technical terms (Handed out to participants in a supplementary sheet).

| *Term* | *Description* |
| --- | --- |
| Total farm work | It includes all types of work directly related to agricultural production (milking, fertilising, etc.) as well as other office and administrative work. Non-agricultural work on a part-time basis is not included in the total farm work. |
| Administrative work | Administrative work includes all tasks related to the application of direct payments. They involve all administrative tasks that are necessary to meet the environmental and animal welfare standards associated with direct payments (e.g. the provision of various forms of documentation in the context of the animal welfare laws and cross-compliance standards). Furthermore, administrative activities include the preparation of documents for on-farm direct payment inspections and the attendance of on-farm inspections. The filing out of complaints following an inspection is also part of the administrative work. |
| Other office work | Other office work includes all tasks that do not take place in the barn or in the field, but are directly related to agricultural production. In addition to farm planning and bookkeeping, this also includes, for example, purchasing and sales, advisory meetings, and further training. However, administrative tasks in the context of the application of direct payments do not count as office tasks. |
